# Effective Method of Purification of Betulin from Birch Bark: The Importance of Its Purity for Scientific and Medicinal Use

**DOI:** 10.1371/journal.pone.0154933

**Published:** 2016-05-06

**Authors:** Pavel Šiman, Alžběta Filipová, Alena Tichá, Mohamed Niang, Aleš Bezrouk, Radim Havelek

**Affiliations:** 1 Charles University in Prague, Faculty of Medicine in Hradec Králové, Department of Medical Biochemistry, CZ-50003, Hradec Králové, Czech Republic; 2 University hospital Hradec Králové, Department of Research and Development, CZ-50005, Hradec Králové, Czech Republic; 3 Charles University in Prague, Faculty of Medicine in Hradec Králové, Department of Medical Biophysics, CZ-50038, Hradec Králové, Czech Republic; University of British Columbia, CANADA

## Abstract

A new and relatively simple method for purification of betulin from birch bark extract was developed in this study. Its five purification steps are based on the differential solubility of extract components in various solvents and their crystallization and/or precipitation, on their affinity for Ca(OH)_2_ in ethanol, and on the affinity of some impurities for silica gel in chloroform. In addition, all used solvents can be simply recycled. Betulin of more than 99% purity can be prepared by this method with minimal costs. Various observations including crystallization of betulin, changes in crystals during heating, and attempt of localization of betulin in outer birch bark are also described in this work. The original extract, fraction without betulinic acid and lupeol, amorphous fraction of pure betulin, final crystalline fraction of pure betulin and commercial betulin as a standard were employed to determine the antiproliferative/cytotoxic effect. We used WST-1 tetrazolium-based assays with triple negative breast cancer cell line BT-549. The decrease in cell survival showed clear relationship with the purity of the samples, being most pronounced using our final product of pure crystalline betulin. WST-1 proliferation/cytotoxicity test using triple negative breast cancer cell line BT-549 clearly showed the importance of purity of betulin for biological experiments and, apparently, for its medicinal use.

## Introduction

**Fig 1:**
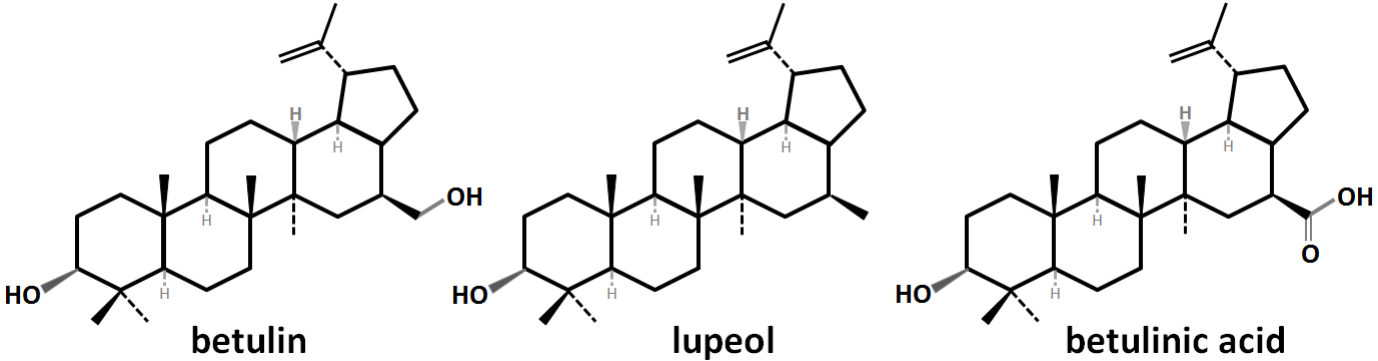
Structure of betulin, lupeol and betulinic acid.

Betulin is a pentacyclic triterpene of lupane type: lup-20(29)-en-3β,28-diol (CAS no. 473-98-3)–see Fig 1. It occurs in a number of plants, especially in many species of birch, where it can be found in large amount in the outer bark. The quantity of betulin can be up to 20–30% (or even nearly 45% [1]) of the dry outer bark weight depending on the tree species and its regional location [2–4]. A lesser amount of betulin can also be found in the root skin and leaves of birches [5].

As a member of the plant pentacyclic triterpene family, betulin has antifungal and antimicrobial effects [6–8]. Thus as with lupeol and betulinic acid, it may protect the tree against fungal and bacterial attack through the bark.

Other triterpenes can be found in birch bark. Lupeol and betulinic acid appear as the most abundant of the other triterpenes. In much lesser amount there may be betulon, erythrodiol and oleanolic acid [9–11]. All the terpenes mentioned above can be easily extracted in the form of a triterpene-rich extract that can be used for biological studies in the same way as the pure substances [10]. The content of betulin and the ratio of triterpenes depend on the botanical species and the locality of the tree [3,4]. The common European species *Betula pendula* Roth., Betulaceae (syn. *B*. *alba*) can contain 10–20% of lupeol and betulinic acid in relation to content of betulin in the outer bark, and both these triterpenes are the main impurities of crude betulin [12]. Lupeol and betulinic acid show similar efficiency in most of their biological activities [13–16]. Moreover, betulin and betulinic acid are valuable templates for many semisynthetic derivatives that can be even more effective drugs [17–22].

It is well known that betulin and other triterpenes exhibit a wide range of important biological effects on animal and human health [5]. Along with the antimycotic and antimicrobial activity mentioned above, anti-inflammatory [23,24], antiviral (including anti-HIV) [16, 20, 25], hepatoprotective [26,27], gastroprotective [28,29], anti-proliferative and anti-cancer [17,23,29–31] properties have previously been demonstrated. Betulin also moderates the biosynthesis of cholesterol and fatty acids, and so ameliorates diet-induced obesity and reduces the size and improves the stability of atherosclerotic plaques (evidenced by reduced accumulation of macrophages) [32]. It can be also used in the treatment of type II diabetes via promotion of insulin sensitivity of cells [32].

Triterpenes may be applied and developed as novel drugs with broad clinical applications [17,20]. Besides this, cosmetic applications have also been reported, and betulin and birch bark extracts are used as additives in cosmetology and food products [21]. Thus, betulin of high purity can be found widely-used in the pharmaceutical and cosmetic industries. Betulin and its semisynthetic derivatives have very high potential for application, mainly in medicine [16,30].

Antiproliferative and/or cytotoxic effect of betulin has been described for many cell lines of human cancers and also on some animal (mouse) cell lines. For most cancer cell lines betulin exhibited cytotoxic potential with IC_50_ (inhibitory effect 50%) values in range 5–10 μg/ml. Only some types of cancer cells resist this treatment and had IC_50_ values about 100 μg/ml and more. It can be summarized that many of malignant cell cultures and cancers are very sensitive to betulin [23,29,31,33].

Moreover betulin is nontoxic compound for whole living organism. The minimal lethal (LD16) and median lethal (LD50) doses in mice are 6500 mg/kg and 9000 mg/kg, respectively [28]. Therefore, according to the international classification, it can be assigned to the 4th class of low-toxic substances [28]. Its low toxicity was documented for experimental animals as mice, rats or dogs. Changes in the ratio of leucocytes and platelets during subchronic toxicity studies was observed after i.p. and s.c. application of more than 100 mg/kg of triterpenoid extract of birch bark (with more than 80% content of betulin) [10]. In pharmacological safety studies this extract showed no histopathological or other deleterious effect at doses up to 540 mg/kg (i.p.) and 300 mg/kg (s.c.), respectively [10].

Birch bark is the best raw material for betulin isolation or production, not only for its content of triterpenes but also for the large amount of bark being produced as waste by the timber industry, especially the paper industry [34]. At present, birch bark is mainly burnt for combined heat and power production instead of its more valuable use as a source of triterpenes, antioxidants and suberin [16,20,35].

Various methods of isolation and purification of betulin have been described, mostly from birch bark. Simple extraction by organic solvents is the easiest way to obtain biologically active material. As solvent we can use methanol, ethanol, propan-2-ol, n-heptane or n-hexane, ethyl acetate and its mixtures with ethanol and water, dichloromethane, a mixture of chloroform/dichloromethane/methanol, a mixture of ethanol and aqueous alkali, butan-1-ol, toluene, petroleum ether, limonene and others [10,12,16,22,23,28,33,36–38]. Ionic liquids based on imidazole are also effective [39]. The extraction is often forced and accelerated by milling or crushing of the bark. Other techniques include ultrasonic disruption and activation of the bark by steam, superheated steam or microwaves [1,11,40,41]. Supercritical extraction with carbon dioxide, with a mixture of methanol, ethanol or acetone, or extraction after esterification of betulin to its diacetate or dipropionate are more difficult methods than simple extraction [1,34,41,42]. A sublimation method has also been described [43], in which atmospheric pressure or high vacuum and high temperatures were used. Recrystallization and/or various column chromatography methods are the major methods for purification of betulin from extracts [22,29,36,37,44,45].

Reverse-phase high performance liquid chromatography (RP-HPLC; [2,3,23,29,38,42,46,47]) and gas chromatography with mass spectrometry detection (GC-MS; [12,48]) are widely used methods for analysis of betulin and other triterpenes in samples. However, thin layer chromatography (TLC, especially in high performance modification—HPTLC) is also well applicable for assessment of purity and visualisation of impurities [9,44,45].

The importance of betulin and other triterpenes in present medicine is obvious and may get an increase. But most purification methods developed so far cannot reach criteria for purity of the drug along with ecological and economical demands. Also the importance of purity of used triterpenes for research tests and for medical applications has not yet been sufficiently supported. This work may give some contribution for solving these problems.

## Materials and Methods

### Ethics Statement

Outer white bark for extraction of betulin was obtained by careful peeling from a relatively freshly felled tree *Betula pendula* in the locality of Hradec Kralove, Czech Republic. The study was carried out on private land and owner of the land gave permission to conduct the study (collection of outer white bark of freshly felled tree *Betula pendula*) on this site. The *Betula pendula* tree is not listed as endangered, protected or rare species.

### Extraction and purification

Notes:

1. A small part of each particular fraction was used for sample analysis and other experimental purposes. Therefore, the amount of fraction used in the next purification step is smaller than the amount of respective fraction obtained in the previous purification step.
2. All fractions mentioned below are marked by letter (B—betulin fraction, L—lupeol fraction, A—betulinic acid fraction) and number (number of purification step; 0—for basic extraction from bark).

#### Extraction

Outer white bark for extraction of betulin was obtained by careful peeling from a relatively freshly felled tree *Betula pendula* in the locality of Hradec Kralove, Czech Republic (coordinates: 50.2429658N, 15.8921664E; spring 2014; botanist: Prof. RNDr. Lubomir Opletal, CSc., Department of Pharmaceutical Botany and Ecology, Faculty of Pharmacy in Hradec Kralove, Charles University in Prague, Czech Republic) on this site. The *Betula pendula* tree is not listed as endangered, protected or rare species. The bark was air-dried indoors in laboratory, shielded from the sun, at room temperature for approximately one month up to constant weight. About 50 g of this bark was cut into small pieces and crushed in a kitchen blender. 300 mL of technical grade ethanol was used for Soxhlet extraction. The bark was extracted only by seven cycles of extraction. The resulting extract was then concentrated by distillation until the first precipitate appeared. This concentrate was cooled to laboratory temperature, and the thick precipitate was filtered on filter glass and quickly washed with cold pure ethanol. Brownish fraction **B0** was obtained after drying the precipitate in air.

#### The first step of purification—removal of betulinic acid

About 0.5 g of freshly precipitated Ca(OH)_2_ obtained from CaCl_2_ and NaOH was first washed with absolute ethanol. This wet hydroxide was then immediately added to a solution of 5 g of fraction **B0** dissolved in 100 mL of hot absolute ethanol. The original brownish colour darkened to brownish-red. This mixture was boiled on a water bath with shaking for about 15 minutes, and the hot precipitate was quickly filtered on filter glass. All the ethanol from the clear brownish-red filtrate was carefully evaporated and the resulting brownish powder of fraction **B1** was dried in air.

The dark brown filter residue of Ca(OH)_2_ with many impurities was washed with cold ethanol and dried. This powder was then treated with 20% HCl to remove hydroxide and the residue—fraction **A1**—was washed with distilled water and dried in air.

#### The second step of purification—removal of lupeol

50 mL of pure benzene was added to 4.7 g of fraction **B1**, stirred and then refluxed for 20 minutes. After cooling to 4°C the resulting gel-like precipitate was centrifuged (30 min., 2500 r.p.m., 4°C) and the supernatant separated and retained. This procedure was repeated twice, and following the third purification by benzene the precipitate was able to be filtrated on a glass filter, and centrifugation was not necessary. After washing of the final precipitate with cold benzene and drying, pale brownish fraction **B2** was obtained.

The three combined benzene fractions (including the benzene washings) were carefully distilled off and nearly all benzene was recycled by distillation and used again for the next purification process of betulin. After evaporation, 1.12 g of a brownish fraction **L2** was obtained.

#### The third step of purification—removal of more polar impurities

In the third step, 3 g of fraction **B2** was dissolved in 120 mL of boiling 96% ethanol and quickly filtered on paper filter (filter for quantitative analysis KA-1-M, Paper mills Pernštejn, Czech Republic). 20 mL of distilled water was then added to the hot filtrate, and the resulting fine precipitate dissolved by boiling to give a clear pale beige solution. This was left to stand overnight at 7°C. The thick precipitate which had formed was centrifuged (30 min., 2500 r.p.m., 4°C) and then dissolved in 70 mL of boiling 96% ethanol. After 5 hours at 7°C the resulting fine crystalline precipitate was filtered on a glass filter, washed with cold ethanol and dried in air. Pale beige fraction **B3** was obtained.

The liquids from the centrifugation and crystallisation procedures were collected and evaporated, and the solid residue was kept for the next isolation as a valuable additive to “future” fraction **B1**.

#### The fourth step of purification—removal of all residual impurities

1 g of fraction **B3** was dissolved in 50 mL of chloroform at room temperature and this clear, slightly yellowish solution was filtered on a column of silica gel (silica gel for column chromatography Silpearl, Glassworks Kavalier, Czech Republic) in chloroform. The height of the column filling was only about 1.5 cm and the diameter of the column was 1 cm. All colored impurities and maybe all residual impurities were visually captured in the top layer of approximately 2–3 mm of the silica gel. The column was then washed with 20 ml of pure chloroform. All eluent was carefully distilled off and bright white amorphous fraction **B4** was obtained.

All the used chloroform—up to 70 mL (except for a very small amount in the silica gel)—was recycled by distillation for use in the next purification process of betulin.

#### The fifth step of purification—crystallization

In the last step, 0.85 g of fraction **B4** was dissolved in 20 mL of boiling absolute ethanol and the clear colourless solution was allowed to stand overnight at 7°C. Betulin crystallized out as flat transparent colourless small crystals, about 0.2 mm length on average. The crystalline precipitate was washed with a small amount of ice-cold absolute ethanol and dried in air. Fraction **B5**, betulin of high purity, was obtained.

**Fig 2:**
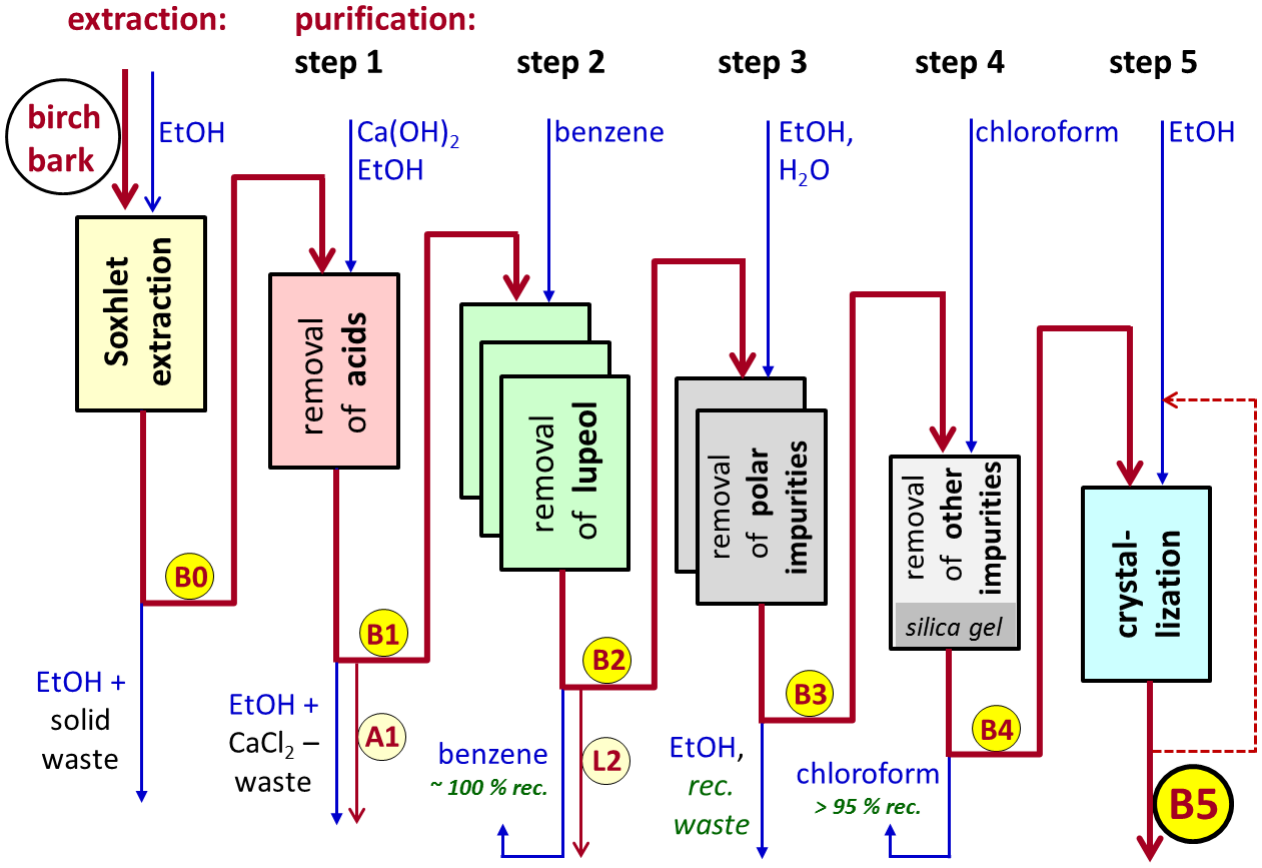
Extraction and purification process.

Extraction and purification of betulin described above is shown in the Fig 2.

### Analysis of samples

Gas chromatography with mass spectroscopy detection was chosen for quantification of the isolated triterpenes. Samples were derivatized with a mixture of pyridine (Sigma Aldrich, USA) with N,O-bis(trimethylsilyl)trifluoroacetamide (Supelco, USA) (1:1 v/v) at 45 min., 75°C. The trimethylsilyl derivatives of the triterpenes were determined by gas chromatography-mass spectrometry (Agilent Technologies—GC 7890A, MS 7890A, USA) and capillary column J and W DB-5 MS 60m x 250 um x 0.25 um (Agilent Technologies, USA). Injector temperature was 280°C and the oven was programmed as follows: initial temperature 70°C, hold time 1 min., rate 15°C/min., to 300°C. The pressure at the column head was 50 kPa. The mass spectrometer was used in electron impact mode (electron energy 70 eV, temperature of source 230°C and quadrupole 150°C). Sample concentrations were quantified using external standard method and this method is resistant to derivatization differences. The ratio of betulin, betulinic acid and lupeol were quantified by calculation of calibration curve.

Betulin of purity >98%, lupeol of purity >94% and betulinic acid of purity >98% from Sigma-Aldrich were used as standards for qualitative determination of the peaks. The standard substances were used for evaluation of retention time of extracts and calculation of amounts of each presented substances. Mass spectra were the same in standards and samples.

### Cell cultures

The WST-1 experiments were carried out with the triple negative breast cancer cell line BT-549 (ATCC, USA). Triple negative breast cancer is a carcinoma negative for estrogen and progesterone receptors and without overexpression of HER/2 protein. We chose this cell line because it would be original research, and also for its high invasiveness as a primary tumor—invasive ductal carcinoma. In the studied publications, betulin has not yet been used for *in vitro* treatment of this cell culture. BT-549 cells were propagated in Dulbecco's Modified Eagle's medium DMEM (Sigma-Aldrich, USA) supplemented with 10% FBS (Life Technologies, USA), 2% glutamine (Life Technologies, UK), 1% penicillin/streptomycin (Life Technologies, UK) and 1% insulin (Sigma-Aldrich, USA). The cell cultures were maintained at 37°C in a humidified incubator in an atmosphere of 5% CO_2_−95% air. BT-549 cells in the maximum range of 20 passages and in an exponential growth phase were used for this study.

### WST-1 proliferation test

Cell proliferation was determined by the WST-1 quantitative colorimetric assay. The solution of betulin was prepared by dissolving 1 mg in 0.5 mL of hot ethanol and 0.5 mL of DMSO. This basic stable solution was then diluted by the complete cultivation medium for BT-549 cells to a concentration of 20 μg/mL (50x), and this final solution was added by programmable microplate dispenser MultiFlo (BioTek Instruments, USA) to minimally the same volume of 24 hours pre-seeded cells in the complete cultivation medium. The 10.000 cells per mL, 400 cells per well in a 384-well plates (Greiner Bio-One, Austria) were treated with betulin at various concentrations from 1 to 10 μg/mL (2.26–22.6 μmol/L) for 24 hours in a humidified atmosphere with 5% CO_2_ and 37°C. After the incubation period, WST-1 was added and the cells were incubated for 3 h. Absorbance of the samples at 440 nm against a background control (medium alone) as a blank was measured using a microplate reader Tecan Infinite M200 (Tecan, Switzerland). The confluence of the cells examined in previous pilot experiments reached under 80% in negative controls over the whole assay time course. Results from cells cultured in medium without betulin were used as a negative control. The results from experiments using doxorubicin treatment for 24 hours at 1 and 2 μM were considered as a positive control. The maximal concentration of ethanol and DMSO in the cell cultures was 0.5%, which is the “safe” non-toxic concentration of both solvents for these cells. This assertion was confirmed experimentally before performing WST-1 tests.

### Statistical analysis

Measurement data were processed and statistically evaluated with the help of MS Excel 2007 (Microsoft Corp, Redmond WA, USA), NCSS 2007 (Hintze, J. (2007). NCSS 2007. NCSS LLC, Kaysville, Utah, USA. www.ncss.com), and GraphPad Prism 5 biostatistics (GraphPad Software, USA). We used Kolmogorov-Smirnov test to test normality of the data distribution. We compared the tested samples with control samples with the help of the two-sample t-test (and Levene’s test to check the homoscedasticity assumption). To adjust for multiple comparisons and keep the family-wise α at 0.05 we used the Bonferroni correction. The resulting α for a single comparison was 0.001. The IC_50_ values of the viability datasets (obtained by use of WST-1 assay) were determined using a non-linear regression.

## Results and Discussion

### Extraction and purification

A relatively simple environmentally friendly chemical method was developed for isolation of betulin from birch bark and its subsequent purification to achieve high-purity (higher than 99%) betulin. Ethanol, water and CaCl_2_ are harmless compounds. Two solvents—benzene and chloroform—are ecologically undesirable, but both are nearly 100% recycled via distillation and can be repeatedly used for further purifications of betulin. NaOH and HCl are used in relatively small amounts and practically in stoichiometric ratio, and therefore are not dangerous for the environment. Solid waste from the bark after extraction is quite environmentally harmless and can be used also as a relatively pure source of suberin. Two of the by-products are a potentially valuable source of betulinic acid (fraction **A1**) and lupeol (fraction **L2**). Taken together, this is why this method can be declared as “green”.

**Fig 3:**
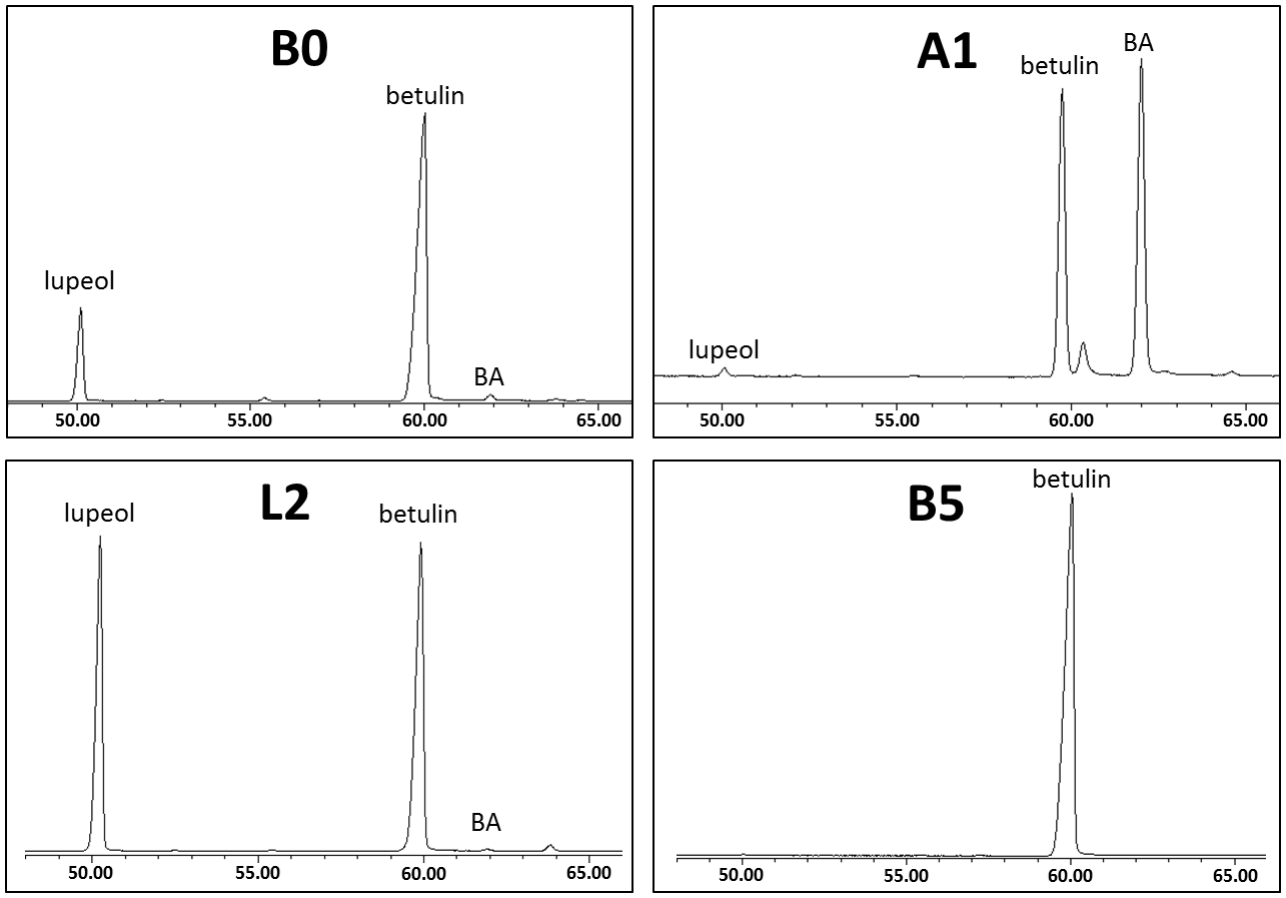
GC-MS chromatograms of four important fractions. **B0**—original extract, **A1**—by-product enriched with betulinic acid, **L2**—by-product enriched with lupeol, **B5**—pure betulin; BA—betulinic acid; relative detector response (Y-axis) against retention time in minutes (X-axis).

The results of extraction and of purification steps are summarized in Table 1. Chromatograms of four most interesting fractions are shown in Fig 3.

**Table 1:** Amount of betulin, betulinic acid, lupeol and other triterpenes in fractions.

	Composition of fractions ^a^				Purification^b^
Fraction	betulin %	BA %	lupeol %	others %	Yield %
**B0**	83.0	1.4	15.2	0.4	(26% of bark)
**B1**	83.5	0.1	15.9	0.5	94
**A1**	45.7	53.3	0.2	0.8	(about 0.7 g)
**B2**	98.2	0.1	1.1	0.6	70
**L2**	55.4	0.2	44.1	0.3	(1.12 g)
**B3**	98.7	0.1	1.0	0.2	43
**B4**	99.2	0.0	0.7	0.1	91
**B5**	**99.8**	0.0	0.2	0.0	71

^a^ Determined by areas of GC-MS chromatogram peaks. Results are shown as % of abundance ratio obtained from calibration curves (BA—betulinic acid).

^b^ Yield of extraction and purification steps in % of the initial amount of the previous fraction.

The total efficiency of the purification process in our case (result: small crystals of fraction **B5**) was about 20% of the initial weight of dry extract **B0**. This “total” efficiency was calculated as a multiple of the efficiencies of the particular purification steps. The real efficiency with respect to the content of betulin in fraction **B0** is somewhat higher. However the betulin from all “wastes” and by-products can be reused in the next purification process as an additive to the initial extract or to solutions of later phases of purification. Theoretically, in a continuous and “endless” (semi)industrial process, the efficiency could reach substantially over 90% of betulin content in the dry extract if no losses on filtration material or on the surfaces of the vessels are considered. Unfortunately, a careful comparison of our method with the methods published in papers referenced earlier [1,10,11,12,16,22,23,28,33,36–45], even considering patents, is not possible as there are insufficient data on efficiency of the extraction and purification process and/or the purity of the product.

The method described in this paper was performed on a laboratory scale only, with gram quantities. However there is no reason why this method is not applicable on a pilot-plant or commercial scale. It is only a matter of the technological equipment and amount of material used. Collectively, all materials are cheap and easily available, including the initial raw material: birch bark, a waste product of the paper and wood industries.

### Crystals

**Fig 4:**
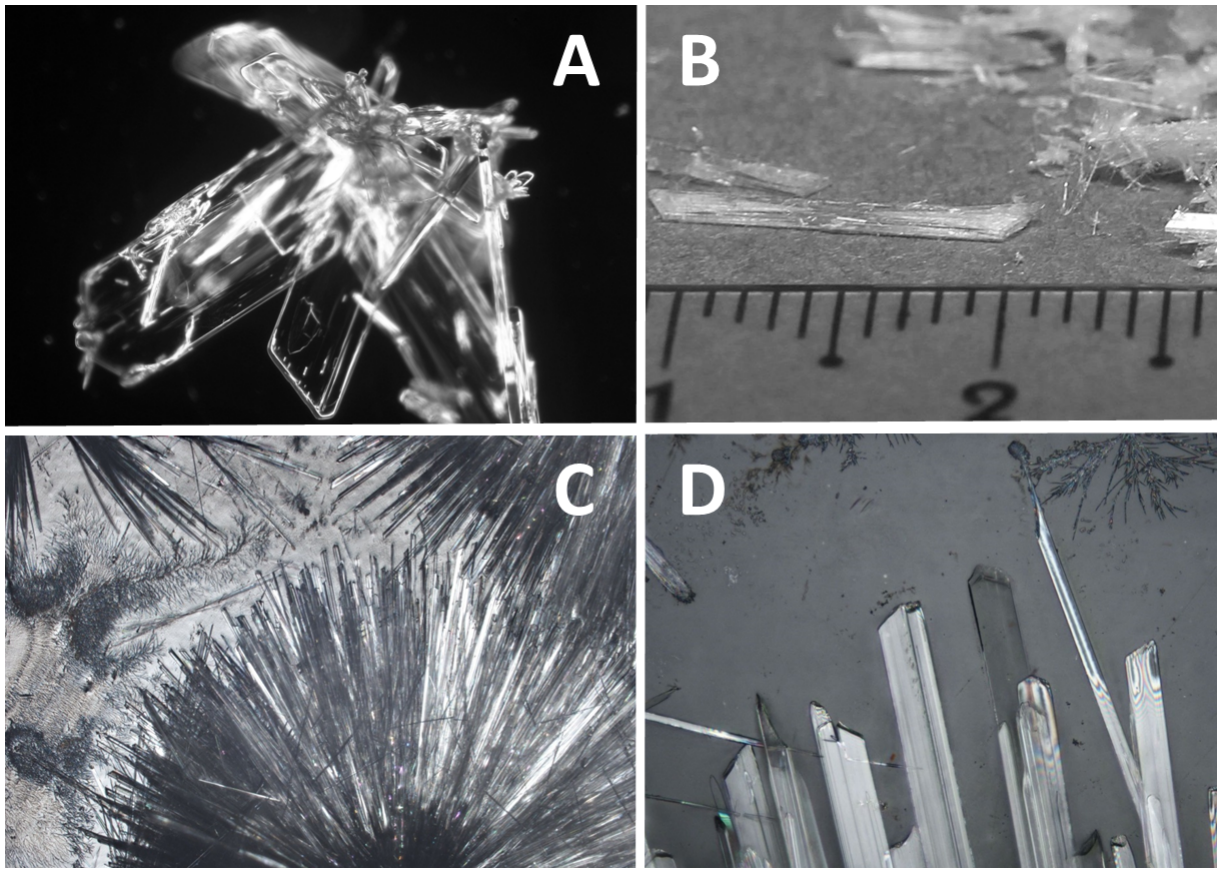
Images of crystals of betulin, fraction B5. A—microphotograph of crystalline fraction; B—large crystals obtained by slow cooling; C and D—macro- and microphotographs of crystals after free evaporation of ethanol.

Fraction **B5** crystallized out the ethanol solution as flat transparent colourless small crystals, about 0.2 mm length on average (see Fig 4A). If the hot saturated ethanolic solution was allowed to cool slowly to room temperature, much bigger crystals formed, up to 12 mm long (Fig 4B). The filtrate was also allowed to stand for spontaneous evaporation in air. Acicular crystals (needle-like, slender crystals) were formed (see Fig 4C—macroscopic photo and 3D—microscopic photo).

Betulin crystallizes from ethanol solution in orthorhombic symmetry with one molecule of ethanol per one molecule of betulin bonded by hydrogen bond [28,49]. Such crystals are quite transparent. About 100 mg of crystals of fraction **B5** were heated to 135°C in a drier for 30 minutes. The resulting brightly white and opaque material was then weighed and from the difference it was estimated that the molecular weight of evaporated material was about 55 Da. If we assume some loss of weight from sublimation of betulin, the result corresponds well with the literature data mentioned above (ethanol: 46 Da).

One of the classic physicochemical characteristics of pure substances is their melting point. Unfortunately, we can find a wide range of melting points assigned to betulin in the literature: 251–261°C [28]. Most authors report a value around 255°C. Pure betulin from Sigma-Aldrich (declared >98% by HPLC) was taken as a standard for comparison. The melting point of the standard was determined at 254–255°C, and for fraction **B5** we measured 255–256°C.

**Fig 5:**
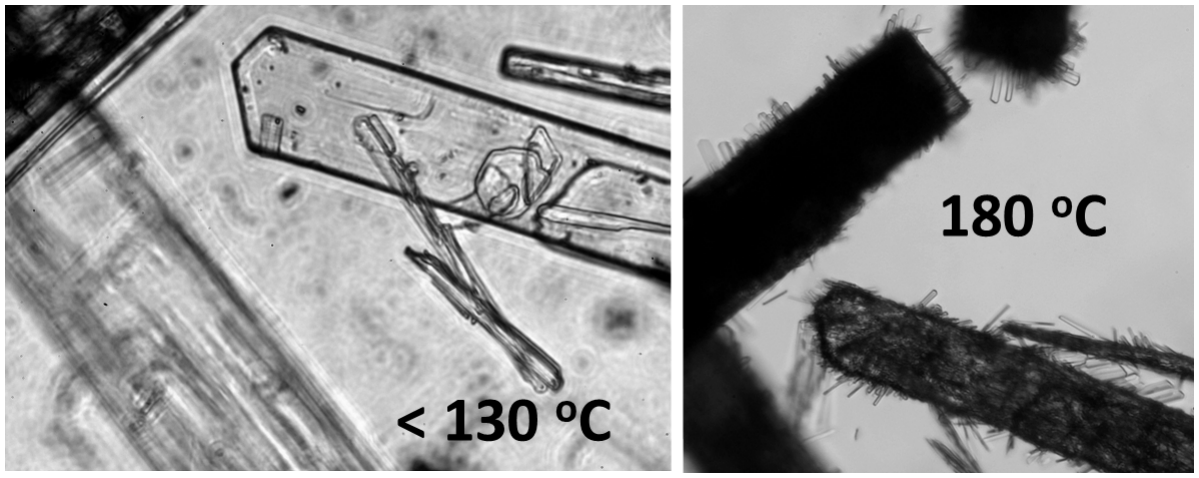
Crystals of fraction B5 during heating.

During heating of a pure **B5** fraction sample under a cover glass changes in the crystals were observed:

130–140°C—darkening of the crystals (in transmitted light in microscope, macroscopically whitening) as crystal-bound ethanol is evaporated, and the crystals visibly fragment (see chapter about crystals above);
160–170°C—betulin begins visibly to sublime;
180–200°C—new transparent microcrystals arise on the surface of the original crystals (see Fig 5);
255–256°C—general melting.

After cooling down no crystals were formed and the melt solidified as an amorphous glass.

Amorphous microscopic beads were also formed as a bright white powder during sublimation under atmospheric pressure in a sublimation apparatus.

### Birch bark

Birch bark consists of brown inner bark ~75% and white outer bark ~25%. The outer bark contains fats, fatty acids, resins, suberin and in particular betulin—up to 30% [20].

**Fig 6:**
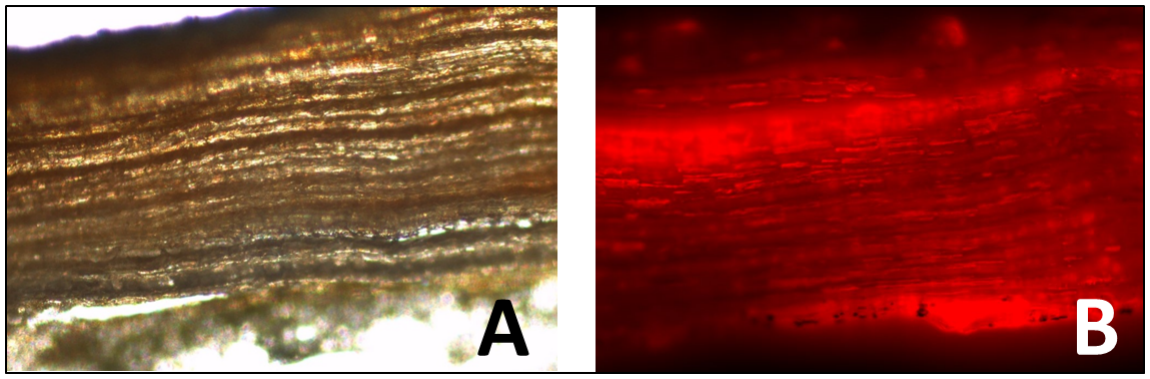
Structure of outer bark. A—bright-field microscopy, B—fluorescence microscopy of outer bark stained by nile red.

The outer bark consists of numerous tightly packed layers of periderm cells on the surface of the stem [50] (see also Fig 6A).

A simple approach for microscopic localization of triterpenes in the outer bark was performed. A lateral cut of the bark was made. Nile red dissolved in dimethyl phthalate was added to this cut and almost immediately was removed again by suction. Fluorescence microscopy was used to obtain a microscopy image (Fig 6B). The fluorescent dye nile red is very lipophilic, and is consequently used for labelling of lipophilic structures, e.g. adipose tissue [51,52]. Thus the brighter regions on the photograph may indicate the locality of hydrophobic triterpenes, especially betulin.

The structure of the outer bark revealed after labelling by nile red may be an interesting result. If mainly triterpenes were labelled by this lipophilic fluorescence dye, we can conclude that betulin is localized in small longitudinal clads through the outer bark. Bearing in mind the existence of triterpene clads and the non-homogeneity of localization of the betulin particles, proper consideration must be given to the mechanical processing of the birch bark before the extraction process. This observation is supported by the fact that when cut into small pieces about 5x5 mm, the bark yielded on average only about 19% extractives, compared with about 26% under the same conditions of extraction after the bark had been crushed in a kitchen blender as described above. Even more so, extraction from paper-like leavings on surface of the bark of thickness about 4 μm gave more than 45% of extractives.

### WST-1 test

**Fig 7:**
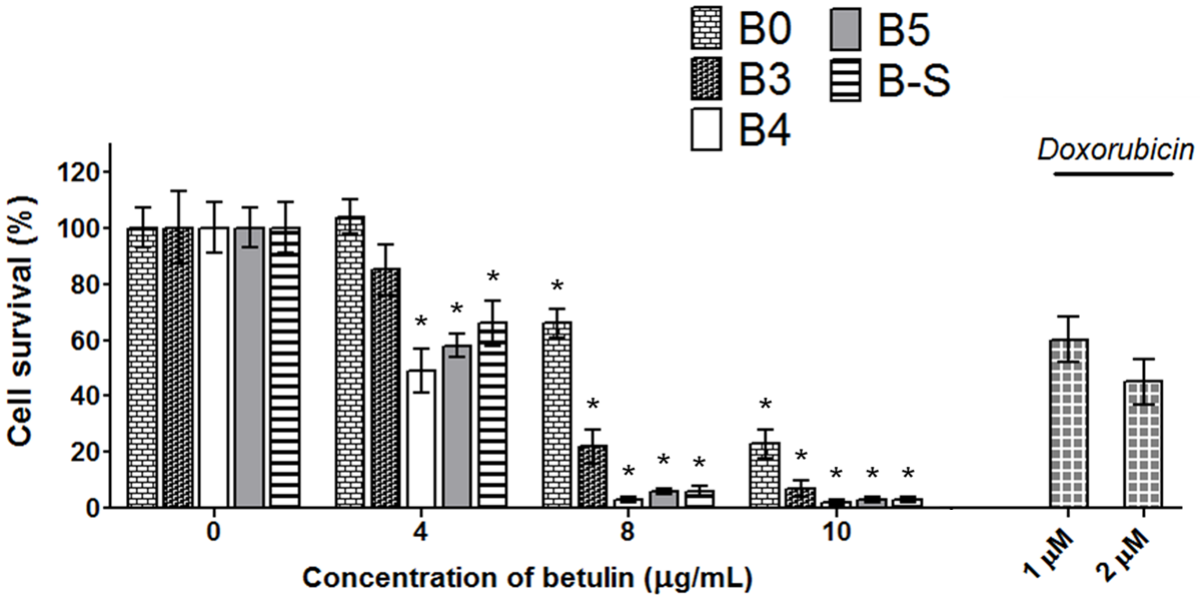
Antiproliferative/cytotoxic effect. **B0**—original extract, **B3**—fraction 3 without betulinic acid and lupeol, **B4**—amorphous fraction of pure betulin, **B5**—crystalline fraction of pure betulin, **B-S**—pure betulin obtained from Sigma-Aldrich. The results are given as relative values to the untreated control in percent. Cells treated with topoisomerase II inhibitor doxorubicin at 1 and 2 μM were used as positive control for decreased cell survival. Bars indicate SD in three independent experiments. *Significantly different from control (P ≤ 0.001).

WST-1 antiproliferation/cytotoxic test in the broad concentration range 1–10 μg/mL was chosen as a control test of biological efficiency of the product. The results of some representative measurements are shown in Fig 7. The points representing concentrations 1 and 2 μg/mL were excluded from data evaluation process using GraphPad statistic software. Betulin in a case of low (subcytotoxic) concentrations, similarly as many other compounds, causes increase in mitochondrial dehydrogenase activities above that of nontreated controls rather than decrease. In our experiment these low concentrations roughly deteriorated accuracy of calculated curves.

The WST-1 test using the human breast cancer BT-549 cell line clearly demonstrated the antiproliferative and/or cytotoxic effect of betulin against this cancer cell line *in vitro*. The results were well reproducible. Four independent experiments showed an IC_50_ value between 4.3–4.6 μg/mL for pure betulin **B5** and 4.6–4.9 μg/mL for standard betulin **B-S** from Sigma-Aldrich. The amorphous fraction **B4** had practically the same IC_50_ value as crystalline fraction **B5** (4.0 and 4.3 μg/mL; two simultaneous measurements). The decrease of BT-549 cell viability compared to the negative controls was statistically significant (P < 0.001) at 3 μg/mL for **B4** (P < 1·10^−6^), **B5** (P < 1·10^−6^) and for **B-S** (P < 0.000564), at 5 μg/mL for **B3** (P < 5·10^−6^), and at 6 μg/mL for **B0** (P < 1·10^−6^). Comparison of effects between the various intermediate products of the purification process showed a clear relationship with the purity of the samples. In accordance with expectation, the original extract **B0** demonstrated the smallest antiproliferative/cytotoxic effect with IC_50_ value nearly doubles that of **B5** (about 9 μg/mL, three measurements). Fraction **B3**—almost pure betulin without betulinic acid and lupeol—had an IC_50_ value also substantially higher (nearly 5.7 and 6.1 μg/mL, two measurements). These results do not exactly correspond to the content of betulin in the fractions and the influence of some inhibitor(s) to betulin efficiency may have to be considered. Such inhibitor(s) possibly remains in the fractions until purification is performed by filtration through silica gel (step 4), and the small rests of colored impurities in the fraction are removed. For example, some antioxidants (phenols, sesquiterpenes and the like) occurred in birch bark [42,48] and in fractions up to fraction **B3** may prevent the cytotoxic effect of betulin. This is one of questions for separate study to explore mode of betulin activity against the cancer cells.

On the basis of these observations we can conclude that the purity of betulin is extremely important for any scientific experiments and measurements in the field of the biological effects of this triterpene. Similarly, the purity may be very important for the medical use of betulin and other triterpenes.

## Conclusions

A relatively simple and cheap method for isolation of betulin from birch bark and its purification to very high purity > 99% was developed. For isolation Soxhlet extraction to ethanol was chosen due to its high efficiency and technical simplicity. The purification procedure consisted of five steps:

1st step—removal of acids and other impurities with Ca(OH)_2_;
2nd step—removal of lupeol by benzene extraction;
3rd step—recrystallization from ethanol solution;
4th step—removal of residual (partly colored) impurities on silica gel in chloroform;
5th step—recrystallization from ethanol solution.

The purification method is environmentally friendly despite the use of benzene and chloroform. Moreover both biologically and environmentally hazardous solvents are almost 100% recycled by distillation and can be reused in further purification processes or used for other purposes. From small (gram-scale for laboratory use) to large industrial-scale amounts of betulin with more than 99% purity can be prepared by this method. On top of this, three valuable by-products are generated—solid bark waste as a potential source of suberin, the fraction after removal of acids as a rich source of betulinic acid, and the fraction after processing by benzene as a rich source of lupeol.

WST-1 proliferation/cytotoxicity test using triple negative breast cancer cell line BT-549 clearly showed the importance of purity of betulin for scientific experiments and for medicinal use. This is an original and high important result.

Various interesting observations regarding pure betulin and birch bark were also described in this work.

## References

[1] Kuznetsova SA, Kuznetsov BN, Skvortsova GP, Vasileva NY, Skurydina ES, Kalacheva GS (2010) Development of the Method of Obtaining Betulin Diacetate and Dipropionate from Birch Bark,. Chemistry for Sustainable Development 18: 265–272.

[2] Hu Z, Guo N, Wang Z, Liu Y, Wang Y, Ding W, et al (2013) Development and validation of an LC-ESI/MS/MS method with precolumn derivatization for the determination of betulin in rat plasma. J Chromatogr B Analyt Technol Biomed Life Sci 939: 38–44. 10.1016/j.jchromb.2013.09.005 24095874

[3] Holonec L, Ranga F, Cranic D, Truta A, Socaciu C (2012) Evaluation of Betulin and Betulinic Acid Content in Birch Bark from Different Forestry Areas of Western Carpathians. Not Bot Horti Agrobo 40: 99–105.

[4] O'Connell MM, Bentleya MD, Campbell CS, Cole BJW (1988) Betulin and lupeol in bark from four white-barked birches. Phytochemistry 27: 2175–2176.

[5] Yin J, Ma H, Gong Y, Xiao J, Jiang L, Zhan Y, et al (2013) Effect of MeJA and Light on the Accumulation of Betulin and Oleanolic Acid in the Saplings of White Birch (*Betula platyphylla* Suk.). American Journal of Plant Sciences 4: 7–15.

[6] Shai LJ, McGaw LJ, Aderogba MA, Mdee LK, Eloff JN (2008) Four pentacyclic triterpenoids with antifungal and antibacterial activity from Curtisia dentata (Burm.f) C.A. Sm. leaves. J Ethnopharmacol 119: 238–244. 10.1016/j.jep.2008.06.036 18662765

[7] Machado KE, Cechinel Filho V, Cruz RC, Meyre-Silva C, Cruz AB (2009) Antifungal activity of Eugenia umbelliflora against dermatophytes. Nat Prod Commun 4: 1181–1184. 19831024

[8] Innocente A, Casanova BB, Klein F, Lana AD, Pereira D, Muniz M, et al (2014) Synthesis of isosteric triterpenoid derivatives and antifungal activity. Chem Biol Drug Des 83: 344–349. 10.1111/cbdd.12251 24138556

[9] E K-B, Duric K, Kalodera Z, Sofic E (2009) Identification and isolation of pharmacologically active triterpenes in *Betulae* cortex, *Betula pendula* Roth., Betulaceae. Bosn J Basic Med Sci 9: 31–38. 19284392 10.17305/bjbms.2009.2853 PMC5645545

[10] Jager S, Laszczyk MN, Scheffler A (2008) A preliminary pharmacokinetic study of betulin, the main pentacyclic triterpene from extract of outer bark of birch (*Betulae alba* cortex). Molecules 13: 3224–3235. 10.3390/molecules13123224 19104487 PMC6245357

[11] Ferreira R, Garcia H, Sousa AF, Freire CSR, Silvestre AJD, Kunz W, et al (2013) Microwave assisted extraction of betulin from birch outer bark. RSC Advances 3: 21285.

[12] Orsini S, Ribechini E, Modugno F, Klügl J, Di Pietro G, Colombini M (2015) Micromorphological and chemical elucidation of the degradation mechanisms of birch bark archaeological artefacts. Heritage Science 3: 2.

[13] Ghaffari Moghaddam M, Ahmad F Bin H., Samzadeh-Kermani A (2012) Biological Activity of Betulinic Acid: A Review. Pharmacology & Pharmacy 03: 119–123.

[14] Saleem M (2009) Lupeol, a novel anti-inflammatory and anti-cancer dietary triterpene. Cancer Lett 285: 109–115. 10.1016/j.canlet.2009.04.033 19464787 PMC2764818

[15] Gallo MBC, M.S J. (2009) Biological Activities of Lupeol. Int J Biomed Pharmac Sci 3: 46–66.

[16] Krasutsky PA (2006) Birch bark research and development. Nat Prod Rep 23: 919–942. 17119640 10.1039/b606816b

[17] Yogeeswari P, Sriram D (2005) Betulinic acid and its derivatives: a review on their biological properties. Curr Med Chem 12: 657–666. 15790304 10.2174/0929867053202214

[18] Melnikova N, Burlova I, Kiseleva T, Klabukova I, Gulenova M, Kislicin CA, et al (2012) A practical synthesis of betulonic acid using selective oxidation of betulin on aluminium solid support. Molecules 17: 11849–11863. 10.3390/molecules171011849 23085649 PMC6268157

[19] Alakurtti S, Makela T, Koskimies S, Yli-Kauhaluoma J (2006) Pharmacological properties of the ubiquitous natural product betulin. Eur J Pharm Sci 29: 1–13. 16716572 10.1016/j.ejps.2006.04.006

[20] Alakurtti S (2013) Synthesis of betulin derivatives against intracellular pathogens VTT Technical Research Centre of Finland: University of Helsinki, Finland 99 p.

[21] Tolstikov GA, Flekhter OB, Shultz EE, Baltina LA, Tolstikov AG (2005) Betulin and Its Derivatives. Chemistry and Biological Activity. Chemistry for Sustainable Development 13: 1–29.

[22] Boryczka S, Bebenek E, Wietrzyk J, Kempinska K, Jastrzebska M, Kusz J, et al (2013) Synthesis, structure and cytotoxic activity of new acetylenic derivatives of betulin. Molecules 18: 4526–4543. 10.3390/molecules18044526 23595090 PMC6270304

[23] Dehelean CA, Soica C, Ledeti I, Aluas M, Zupko I, Galuscan A, et al (2012) Study of the betulin enriched birch bark extracts effects on human carcinoma cells and ear inflammation. Chem Cent J 6: 137 10.1186/1752-153X-6-137 23158079 PMC3527166

[24] Recio MC, Giner RM, Manez S, Rios JL (1995) Structural requirements for the anti-inflammatory activity of natural triterpenoids. Planta Med 61: 182–185. 7753929 10.1055/s-2006-958045

[25] Gong Y, Raj KM, Luscombe CA, Gadawski I, Tam T, Chu J, et al (2004) The synergistic effects of betulin with acyclovir against herpes simplex viruses. Antiviral Res 64: 127–130. 15498608 10.1016/j.antiviral.2004.05.006

[26] Miura N, Matsumoto Y, Miyairi S, Nishiyama S, Naganuma A (1999) Protective effects of triterpene compounds against the cytotoxicity of cadmium in HepG2 cells. Mol Pharmacol 56: 1324–1328. 10570061 10.1124/mol.56.6.1324

[27] Szuster-Ciesielska A, Kandefer-Szerszen M (2005) Protective effects of betulin and betulinic acid against ethanol-induced cytotoxicity in HepG2 cells. Pharmacol Rep 57: 588–595. 16227641

[28] Kuznetsova SA, Skvortsova GP, Maliar IN, Skurydina ES, Veselova OF (2014) Extraction of betulin from birch bark and study of its physico-chemical and pharmacological properties. Russian Journal of Bioorganic Chemistry 40: 742–747.

[29] Drag M, Surowiak P, Drag-Zalesinska M, Dietel M, Lage H, Oleksyszyn J (2009) Comparision of the cytotoxic effects of birch bark extract, betulin and betulinic acid towards human gastric carcinoma and pancreatic carcinoma drug-sensitive and drug-resistant cell lines. Molecules 14: 1639–1651. 10.3390/molecules14041639 19396022 PMC6254329

[30] Şoica C (2012) Betulin—A Future Key-Player in the Treatment of Neoplasic Diseases. Medicinal & Aromatic Plants 01.

[31] Krol SK, Kielbus M, Rivero-Muller A, Stepulak A (2014) Comprehensive review on betulin as a potent anticancer agent. Biomed Res Int 2015: 584189. 10.1155/2015/584189 PMC4383233 25866796

[32] Tang JJ, Li JG, Qi W, Qiu WW, Li PS, Li BL, et al (2011) Inhibition of SREBP by a small molecule, betulin, improves hyperlipidemia and insulin resistance and reduces atherosclerotic plaques. Cell Metab 13: 44–56. 10.1016/j.cmet.2010.12.004 21195348

[33] Soica CM, Dehelean CA, Peev C, Aluas M, Zupko I, Kasa P, et al (2012) Physico-chemical comparison of betulinic acid, betulin and birch bark extract and *in vitro* investigation of their cytotoxic effects towards skin epidermoid carcinoma (A431), breast carcinoma (MCF7) and cervix adenocarcinoma (HeLa) cell lines. Nat Prod Res 26: 968–974. 10.1080/14786419.2010.545352 21598174

[34] Šarek J, Svoboda M, Hajduch M (2012) Method of preparation and isolation of betulin diacetate from birch bark from paper mills and its optional processing to betulin.

[35] Ferreira R, Garcia H, Sousa AF, Freire CSR, Silvestre AJD, Rebelo LPN, et al (2013) Isolation of suberin from birch outer bark and cork using ionic liquids: A new source of macromonomers. Industrial Crops and Products 44: 520–527.

[36] Lugemwa FN (2012) Extraction of betulin, trimyristin, eugenol and carnosic acid using water-organic solvent mixtures. Molecules 17: 9274–9282. 10.3390/molecules17089274 22864237 PMC6268899

[37] Boryczka S, Michalik E, Jastrzebska M, Kusz J, Zubko M, Bebenek E (2012) X-Ray Crystal Structure of Betulin–DMSO Solvate. Journal of Chemical Crystallography 42: 345–351.

[38] Zhao G, Yan W, Cao D (2007) Simultaneous determination of betulin and betulinic acid in white birch bark using RP-HPLC. J Pharm Biomed Anal 43: 959–962. 17084057 10.1016/j.jpba.2006.09.026

[39] Ressmann AK, Strassl K, Gaertner P, Zhao B, Greiner L, Bica K (2012) New aspects for biomass processing with ionic liquids: towards the isolation of pharmaceutically active betulin. Green Chemistry 14: 940.

[40] Chen QH, Fu ML, Liu J, Zhang HF, He GQ, Ruan H. (2009) Optimization of ultrasonic-assisted extraction (UAE) of betulin from white birch bark using response surface methodology. Ultrason Sonochem 16: 599–604. 10.1016/j.ultsonch.2008.11.009 19110462

[41] Mikhailenko MA, Shakhtshneider TP, Drebushchak VA, Kuznetsova SA, Skvortsova GP, Boldyrev VV (2011) Influence of mechanical treatment on the properties of betulin, betulin diacetate, and their mixture with water-soluble polymers. Chemistry of Natural Compounds 47: 229–233.

[42] Co M, Koskela P, Eklund-Åkergren P, Srinivas K, King JW, Sjöberg PJR, et al (2009) Pressurized liquid extraction of betulin and antioxidants from birch bark. Green Chemistry 11: 668.

[43] Guidoin M-F, Yang J, Pichette A, Roy C (2003) Betulin isolation from birch bark by vacuum and atmospheric sublimation. A thermogravimetric study. Thermochimica Acta 398: 153–166.

[44] Joshi H, Saxena GK, Singh V, Arya E, Singh RP (2013) Phytochemical Investigation, Isolation and Characterization of Betulin from Bark of *Betula Utilis*. J Pharmacognosy Phytochem 2: 145–151.

[45] Tijjani A, Ndukwe IG, Ayo RG (2012) Isolation and Characterization of Lup-20(29)-ene-3, 28- diol (Betulin) from the Stem-Bark of Adenium obesum (Apocynaceae). Tropical Journal of Pharmaceutical Research 11.

[46] Maji AK, Maity N, Banerji P, Banerjee D (2013) Validated RP-HPLC-UV method for the determination of betulin in Asteracantha longifolia (L.) Nees. extract. Int J Phytomed 5: 131–135.

[47] Pfarr K, Danciu C, Dehelean C, Pfeilschifter JM, Radeke HH (2014) Betulin—a plant-derived cytostatic drug—enhances antitumor immune response. Journal for ImmunoTherapy of Cancer 2: P175.

[48] Regert M, Alexandre V, Thomas N, Lattuati-Derieux A (2006) Molecular characterisation of birch bark tar by headspace solid-phase microextraction gas chromatography-mass spectrometry: a new way for identifying archaeological glues. J Chromatogr A 1101: 245–253. 16236293 10.1016/j.chroma.2005.09.070

[49] Drebushchak TN, Mikhailenko MA, Brezgunova ME, Shakhtshneider TP, Kuznetsova SA (2010) Crystal Structure Of Betulin Ethanol Solvate. Journal of Structural Chemistry 51: 798–801.

[50] Bhat KM (1982) Anatomy, Basic Density and Shrinkage of Birch Bark. IAWA Bull 3: 207–211.

[51] Greenspan P, Fowler SD (1985) Spectrofluorometric studies of the lipid probe, nile red. J Lipid Res 26: 781–789. 4031658

[52] Greenspan P, Mayer EP, Fowler SD (1985) Nile red: a selective fluorescent stain for intracellular lipid droplets. J Cell Biol 100: 965–973. 3972906 10.1083/jcb.100.3.965 PMC2113505

